# Rehabilitation During COVID-19 Pandemic: An Indian Perspective

**DOI:** 10.1017/dmp.2020.316

**Published:** 2020-09-02

**Authors:** Harleen Uppal, Siddharth Rai

**Affiliations:** Department of Physical Medicine and Rehabilitation, Dr Baba Saheb Ambedkar Medical College and Hospital, Rohini, New Delhi, India; Department of Physical Medicine and Rehabilitation, Apex Trauma Centre, Sanjay Gandhi Post graduate Institute of Medical Sciences, Lucknow, India

**Keywords:** COVID-19, neurological rehabilitation, pulmonary rehabilitation, rehabilitation, SARS-CoV-2, tele-rehabilitation

## Abstract

It has been noted that as high as 20.3% of patients hospitalized for coronavirus disease 2019 (COVID-19) require intensive care unit (ICU) admission. This has most commonly been attributed to the development of acute respiratory distress syndrome. These patients require prolonged periods of ICU stay, averaging approximately 20 days. As people recover and are discharged, there will be a new pandemic of critical illness survivors. These patients would present with impairments and disabilities arising because of prolonged ICU stay as well as consequences of severe respiratory illness. The longer the duration of ICU stay, the higher is the risk for long-term physical, cognitive, and emotional impairments needing comprehensive and early rehabilitation. This article focuses on the indispensable role of early and interdisciplinary rehabilitation in effective disaster management, restoring functions, and improving quality of life in COVID survivors. It outlines how to practically expand rehabilitation services in a resource-limited country, such as India, and lists the limitations being faced that prevent the uniform application of rehabilitation services in India. This would help to deal with the rapid increase in demand of postacute care facilities, be it in hospital services, in the form of inpatient or outpatient rehabilitation or home care facilities, including telemedicine.

Coronavirus disease 2019 (COVID-19) is a potentially serious acute respiratory infection caused by severe acute respiratory syndrome coronavirus 2 (SARS-CoV-2).^1^ It is alarming to note that, 20.3% of those hospitalized for COVID-19 need intensive care unit (ICU) admission, mostly due to development of acute respiratory distress syndrome. (ARDS) (32.8%).^2^

In the weeks and months following the surge in cases, there will be a rapid increase in the number of COVID-19 survivors, some of whom would have spent a long time immobilized in the ICU, battling critical illness and requiring rehabilitation. Hospitals should be well equipped with rehabilitation professionals to cater to the acute needs of survivors being discharged from the ICUs. An early rehabilitation intervention will help in speeding up recovery from residual impairments, maintaining continuity of care, improving quality of life, and overall functional outcome. The presence of physiatrists and the Physical Medicine and Rehabilitation (PM&R) Department will decongest the acute services, creating space for newly affected patients to receive acute care. In short, they will make the hospital services more efficient by decreasing the length of ICU stay and providing a proper outlet for acute services. This fact is further reinforced by the World Health Organization (WHO) Emergency Medical Team minimum standards, which recommends that rehabilitation should be a core component of patient centered care while responding to disasters.^3^

Unfortunately, in India as well, facilities for postacute and rehabilitation care are grossly lacking and are being given less priority. The purpose of this review is to explain the role of interdisciplinary rehabilitation during this pandemic and how it helps in restoring functions and improving quality of life in COVID survivors.

## COVID-19: CLINICAL PRESENTATION

Broadly, SARS-CoV-2 syndrome can be divided into the following 2 phases: the acute phase having predominantly respiratory symptoms, and the chronic phase comprising of sequelae of prolonged ICU stay, invasive mechanical ventilation, and prolonged immobilization.

Recent literature suggests that COVID-19 virus also affects the central nervous system per se. Patients having more severe respiratory symptoms have a higher rate of neurological symptoms.^4^ A study from Wuhan has stated that around 36.4% patients demonstrated neurological conditions.^4^ Hospitals in Italy have reported many patients suffering from vascular events, such as ischemic strokes and thrombosis, as a consequence of virus targeting the coagulating mechanisms.^5^

## COVID-19: SEQUELAE

Critical illness influences a wide range of long-term patient outcomes, with some impairments persisting beyond the ICU stay, resulting in chronic disability leading to post-intensive care syndrome (PICS).^6^

COVID-19 patients, who are either admitted to the ICU for severe respiratory failure or have neurological symptoms, are more predisposed to develop infection associated chronic neuroinflammation and brain cell degeneration leading to long-term neurological sequelae.^7^ To support this fact, it has been shown that one-third of COVID-19 patients at discharge have motor and cognitive symptoms, such as inattention, disorientation, and poor movements in response to commands.^8^ Many neuropsychiatric complications have been documented in COVID-19 patients. Higher blood levels of pro-inflammatory cytokines have led to development of encephalopathies. Depression, anxiety, and posttraumatic stress disorder have also been seen.^9^

## COVID-19: ROLE OF REHABILITATION

The WHO has defined medical rehabilitation as “a set of interventions designed to optimize functioning and reduce disability in individuals with health conditions, in interaction with their environment”. It has been recommended that physiatrists should be included in the interdisciplinary teams for acute management as well as management of sequelae of COVID-19.^10^ Physiatrists effectively integrate and coordinate patients’ requirements in the context of rehabilitation and clinical needs with other health-care providers.^10^ The potential role of physiatrists are early management of impairments and disabilities, improving functional capabilities, recommend evidence-based practice approach, preventing complications, and reintegrating patients back into society.^11^

A multidisciplinary care model should be followed to prevent PICS, known as the ABCDE bundle. Components of the ABCDE bundle are awakening (using light or minimal sedation), breathing (spontaneous breathing trials), coordination of care and communication among various disciplines, delirium monitoring, assessment & management and early ambulation in the ICU.^12^

As patients with serious COVID-19 infection usually have other comorbidities, such as advanced age, multiple chronic ailments, obesity, and multiple organ failure, a tailor-made rehabilitation plan should be made for each patient, focusing on each impairment.

The goals of rehabilitation for recovery of COVID-19 patients can be outlined as: (a) Improve pulmonary function, (b) Reverse side effects of prolonged Immobilization, (c) Improve cognitive functions and management of dysphagia, and (d) Reduce impairments and disabilities.

## CURRENT INDIAN SCENARIO AND PM&R INTERVENTIONS

Rehabilitation of COVID-19 patients can be categorized broadly into mildly symptomatic and severely symptomatic patients. The majority of patients in India are suffering from mild symptoms and are being managed in home isolation or at quarantine facilities made available by the government. Teleconsultation services have been made available by various government institutes, through which doctors can keep in touch with the COVID-19 positive patients being isolated at home, and refer them to a hospital in case the patient is a high-risk patient with red flags. The general public has been informed about the new telemedicine services through print and digital media. Many private hospitals have also offered packages, including regular monitoring of vitals and daily teleconsultations with doctors. Mental Health Services in the form of teleconsultation has been made available for health-care workers by the Government of India.

Patients who are severely ill require acute hospitalization. The rehabilitation management of severely symptomatic COVID-19 patients can be categorized into 2 phases. The first phase also known as acute phase, incorporates multidisciplinary management of noninvasive ventilation along with continuous monitoring of clinical parameters and signs of infections. Pulmonary rehabilitation is not recommended for patients in acute stage of ARDS and unstable patients except for positioning advice and range of motion exercises.^13^ Once the patient’s symptoms have become stable, ie, fever has decreased, dyspnea improved, having respiratory rate of < 30 breaths/min, oxygen saturation (SpO_2_) > 90%, an individualized rehabilitation plan appropriate to the specific impairments of the patient should be formulated. This plan incorporates supporting pulmonary hygiene, training of bronchial clearance techniques in hypersecretive patients, and deep coughing into closed plastic bags for sputum collection to prevent any spread of virus, and teaching breathing techniques. Airway clearance techniques are not required if patient has dry cough.^13^

For disability prevention, this phase of rehabilitation includes optimizing bed positioning, active-assisted or active joint mobilization, whole body muscle strengthening and frequent changes of posture. Gradually increasing the anti-gravity position based on the patient’s clinical condition until the patient can maintain an upright position, plays an indispensable role. A semi-sitting or sitting position is recommended to prevent the risk of aspiration. Prolonged prone ventilation, even more than 12 h/d is a very advantageous position for those suffering from ARDS.^14^ Prone positioning is advantageous as it improves the ventilation perfusion ratio at the most severely affected areas of lung, that is, the basilar or peripheral regions of the lungs. Recovery of lost motor function by early mobilization and promoting activity mainly includes only bedside activities in this phase. It is recommended that mobilization should be encouraged as soon as possible with sitting in bed followed by sitting at the edge of the bed, transfer to the chair, and standing with the help of a tilt table.^13^ All these techniques and exercises, deemed appropriate by PM&R specialist, should be taught to the patient by 1 of the rehabilitation team members (physiotherapist, occupational therapist, or nurse) during a single session.^13^ Where ever possible, videos, instructional manuals, and remote consultations can also be used for demonstration and education purposes.

Those patients having significant pulmonary and neuromotor symptoms, even after discharge from the ICU, are transferred to COVID-19 wards. Patients having Functional Independence Measure (FIM) scores of less than 100 with dependence in at least 2 areas,^14^ should undergo structured rehabilitation programs in the inpatient PM&R wards to improve the chances of recovery, while those cases with few and minor sequelae of COVID-19 infection may undergo home or outpatient rehabilitation therapy. This phase comprises of second phase of rehabilitation. The management in this second phase focusses on reducing the risk of aspiration, which can be due to cognitive deficits, brainstem abnormality, etc. Patient should be advised of appropriate diet modification to prevent the risk of aspiration. This also includes aerobic exercises for physical deconditioning, passive/active mobilization, muscle strength training, static and dynamic balance training for balance dysfunction, and joint proprioceptive training.^15^ Furthermore, patients can be evaluated for the ability to carry out activities of daily living and guided to improve recovery and adaptation to them. Other clinical issues, such as management of postintubation iatrogenic dysphagia, deep venous thrombosis prophylaxis, prevention of pressure ulcers, optimizing bladder and bowel management, prevention of osteoporosis, regulating sleep wake cycle, as well as management of neuropsychological and cognitive issues, are also addressed in this phase of multidisciplinary rehabilitation.

Neuropsychological support in the form of counselling sessions, and cognitive training, such as cognitive stimulation, reorientation, etc., should be included in the rehabilitation programs of such patients. Here, it is appropriate to point out that all the components of this comprehensive inpatient rehabilitation need to be undertaken at bedside with mandatory personal protective equipment. Due to indigenous manufacturing of personal protective equipment (PPE), this should not be a limiting factor in India, as it is in some other countries.

## OUTPATIENT REHABILITATION & TELE-REHABILITATION IN INDIA

The safest mode of rehabilitation for delivering outpatient services is virtual rehabilitation or tele-rehabilitation. It minimizes contact and viral spread, and provides timely rehabilitation care amid distance. In India, media applications, such as WhatsApp, and other teleconsultation software, such as Hospital Information System, are being effectively used. Many mobile applications, such as Practo and Lybrate, have also become quite popular in providing teleconsultations in private practice. As of now these services are gaining a lot of popularity and are proving to be very useful for the patients. Virtual rehabilitation can be helpful in critical care services in ICU, wherein, whoever has been admitted with COVID in the ICU for more than 7 days, can have a virtual consultation with a physiatrist for rehabilitation service evaluation. This avenue needs to be further explored in the Indian setting. These patients, once discharged, should be followed up by tele-rehabilitation service or in-person out-patient department consultations in rehabilitation clinics, for assessing pulmonary, cognitive, behavioral, physical, balance, dysphagia issues, and so that conditions, such as PICS, are not missed.

However, there are many limitations of tele-rehabilitation, such as availability of equipment and technology, especially in the rural areas. India has a vast majority of population who are poor and cannot afford a smart phone or a laptop. Other factors, such as lack of knowledge and skills to use technology, limited Internet services in the periphery, lack of satisfaction, and lack of tele-rehabilitation guidelines, serve as barriers to the efficient application of tele-rehabilitation in India uniformly.

## POTENTIAL BARRIERS

However, there is a need for post discharge care and rehabilitation of those cured of the disease to be incorporated into the system. In India, apart from its huge population and poor doctor to population ratio, lack of PM&R departments and staffing in many hospitals, in both government and private setups are major challenges. Most importantly, lack of government policies directed toward rehabilitation and lack of awareness of government and general public about the need and importance of rehabilitation of COVID-19 patients are major barriers in achieving rehabilitation of COVID-19 survivors.

## CONCLUSIONS

Physiatrists can help in the early recovery of patients with COVID-19 by delivering early rehabilitation starting from the ICU and throughout the acute hospital stay. For this, interdisciplinary rehabilitation should be initiated, which can be continued after discharge from the ICU to either inpatient rehabilitation in a specialized Rehabilitation Medicine wards or an outpatient setting, using either in-person or virtual rehabilitation.
